# Sensory impairments and epigenetic aging: insights from self-rated hearing and vision in United States adults

**DOI:** 10.1007/s11357-025-01706-6

**Published:** 2025-05-23

**Authors:** Jamaji C. Nwanaji-Enwerem, Dennis Khodasevich, Nicole Gladish, Hanyang Shen, Anne K. Bozack, Saher Daredia, Belinda L. Needham, David H. Rehkopf, Andres Cardenas

**Affiliations:** 1 Department of Emergency Medicine, Center for Health Justice, and Center of Excellence in Environmental Toxicology, Perelman School of Medicine, University of Pennsylvania, Ground Ravdin, HUP, 3400 Spruce Street, Philadelphia, PA 19104 USA; 2 Department of Epidemiology and Population Health, Stanford University, Palo Alto, CA USA; 3 Division of Epidemiology, UC Berkeley School of Public Health, Berkeley, CA USA; 4 Department of Epidemiology, Center for Social Epidemiology and Population Health, School of Public Health, University of Michigan, Ann Arbor, MI USA

**Keywords:** DNA methylation age, NHANES, Auditory, Visual, Disability

## Abstract

Sensory impairments are common with aging, but studies examining the relationships of these impairments with DNA methylation–based biomarkers of aging, strong predictors of morbidity and mortality, remain sparse. We investigated whether subjective measures of sensory impairment are associated with epigenetic age biomarkers. We conducted a cross-sectional analysis in a representative sample of 2344 U.S. adults from the 1999–2000 and 2001–2002 cycles of the National Health and Nutrition Examination Survey (NHANES). We examined the relationships of self-rated auditory and vision function with seven epigenetic aging biomarkers: HannumAge, HorvathAge, SkinBloodAge, PhenoAge, GrimAge2, DNA methylation telomere length, and DunedinPoAm. We adjusted for potential confounders including chronological age, other demographics, lifestyle factors, and general health. In adjusted survey-weighted models, self-reported deafness was associated with a significantly higher GrimAge2 (*β* = 4.19-years, 95% CI 2.29, 6.09, *P* = 0.004) and DunedinPoAm (*β* = 0.07, 95% CI 0.04, 0.09, *P* = 0.002) compared to good hearing. Deafness was also associated with significantly higher GrimAge2 estimates of TIMP1 (*β* = 459.51, 95% CI 287.00, 632.03 *P* = 0.002) and marginally higher estimated levels of ADM (*β* = 10.06, 95% CI 1.76, 18.36, *P* = 0.03), CRP (*β* = 0.34, 95% CI 0.11, 0.56, *P* = 0.01), and cigarette pack-years (*β* = 6.55, 95% CI 2.62, 10.47, *P* = 0.01). No associations were observed with self-rated vision. We describe associations of self-rated deafness with accelerated epigenetic aging, as measured by GrimAge2 and DunedinPoAm. These results provide a foundation for future research exploring epigenetic biomarkers as tools for predicting and understanding the biological processes underlying sensory impairments like deafness.

**Supplementary Information:**

The online version contains supplementary material available at 10.1007/s11357-025-01706-6.

## Introduction

Studies estimate that up to 94% of older United States (U.S.) adults experience at least one sensory deficit [1]. Among these, hearing and vision impairments are the most prevalent and can significantly impact daily functioning, reducing independence, diminishing quality of life, and increasing the risk of morbidity (e.g., cognitive decline) and mortality [2–6]. Given their widespread prevalence and profound impact on daily life [2, 3, 7, 8], understanding the biological processes involved in hearing and vision impairments remains a public health priority.

One promising avenue for investigating these processes is through relationships with epigenetic aging, DNA methylation–based biomarkers of age that have been linked to disease risk, as well as environmental and social exposures [9–15]. While previous research has linked epigenetic aging to auditory and vision pathology, these studies have been limited in sample size and scope. For instance, an analysis of the Baltimore Longitudinal Study of Aging cohort found that worse hearing (higher speech-frequency pure tone average) was associated with GrimAge and DunedinPoAm acceleration, but not other epigenetic aging biomarkers such as HannumAge, HorvathAge, and PhenoAge [16]. Among the most notable reported vision impairment findings include associations of HorvathAge acceleration with increased risk of glaucoma in a European ancestry study sample [17], and associations of HorvathAge and HannumAge acceleration with the progression of glaucoma in a study sample made up of Black and White participants from North Carolina, U.S. [8].

The present study expands on previous research by leveraging data from the National Health and Nutrition Examination Survey (NHANES), a nationally representative sample of the U.S. population, to examine the cross-sectional relationships of self-rated hearing and vision function with epigenetic age in adults aged 50 years and older. An important consideration that is specifically addressed by this study is whether self-rated assessments of auditory and visual function align with relationships previously reported with more objective measurements. Given the well-established link of hearing and vision impairments with chronological aging [3, 7], we will also compare the associations we observe between sensory function and epigenetic age with the results of models examining the associations between sensory function and chronological age. Given that epigenetic age is sensitive to age-related pathology [18], we hypothesize that hearing and vision impairments will be associated with greater epigenetic aging. Furthermore, because epigenetic aging is considered a more sensitive indicator of morbidity [18], we anticipate that observed associations with epigenetic age will be stronger than those observed with chronological age.

## Methods

### Study population

The National Center for Health Statistics (NCHS) conducts the National Health and Nutrition Examination Survey (NHANES) to evaluate the health of the noninstitutionalized U.S. population using data collected from NHANES through structured interviews, physical examinations, and laboratory tests. This study utilized publicly available data from the 1999–2000 and 2001–2002 NHANES cycles to investigate the associations of self-rated hearing and vision with epigenetic age.

The initial study sample included 2532 adults aged 50 years and older. NHANES top-coded the ages of participants aged 85 years and older as 85 years to safeguard participant privacy (*n* = 130). Because the exact chronological ages were not available for these participants, they were excluded from the analysis to avoid potential misclassification errors in epigenetic age measurements. Additionally, we excluded participants whose DNA methylation (DNAm)-predicted sex did not match their self-reported sex (*n* = 56) to ensure data accuracy. After these exclusions, the final analytical sample consisted of 2346 participants. Among these participants, 2344 had complete data on self-rated hearing and 2317 participants had complete data on self-rated vision. A detailed flowchart of the sample selection process is provided in Figure S1. All participants provided written informed consent, and the study protocols were approved by the NCHS Research Ethics Review Board (protocol #98–12).

### Self-rated hearing and vision

As part of the audiometry questionnaire, participants were asked to rate their hearing by responding to the question, “Which statement best describes your hearing (without hearing aid)? Would you say your hearing is good, that you have a little trouble, a lot of trouble, or are you deaf?” (https://wwwn.cdc.gov/Nchs/Data/Nhanes/Public/1999/DataFiles/AUQ.htm). As part of the vision questionnaire, participants were also asked to rate their vision with the question, “At the present time, would you say your eyesight, with glasses or contact lenses if you wear them, is…” with options of: “Excellent,” “Good,” “Fair,” “Poor,” or “Very Poor” (https://wwwn.cdc.gov/Nchs/Data/Nhanes/Public/1999/DataFiles/VIQ.htm). Those who declined to answer the questions, were uncertain about their hearing/vision status, or had missing data were excluded from the analysis.

### DNA methylation and epigenetic age

We downloaded epigenetic age measures and DNA methylation–based leukocyte proportion estimates from the NHANES website (https://wwwn.cdc.gov/nchs/nhanes/dnam/), which includes comprehensive details on DNA methylation analysis and processing. In brief, DNA was extracted from whole blood samples collected from NHANES participants aged 50 years and older during the 1999–2000 and 2001–2002 cycles. Genome-wide DNA methylation was subsequently analyzed using the Illumina EPIC BeadChip array, following the manufacturers’ protocol and standard quality control procedures.

This study incorporated seven epigenetic age measures: HannumAge, HorvathAge, SkinBloodAge, PhenoAge, GrimAge2, DunedinPoAm, and DNA methylation-based telomere length (DNAmTL). These measures were selected a priori due to their well-documented associations with health outcomes [9–14, 19, 20]. HannumAge, HorvathAge, and SkinBloodAge were developed to predict chronological age in years based on DNA methylation patterns, though they have also been linked to broader health indicators [10, 11, 20]. PhenoAge, a biomarker of healthspan, was constructed using a composite of nine clinical variables: albumin, creatinine, glucose, C-reactive protein, lymphocyte percentage, mean cell volume, red cell distribution width, alkaline phosphatase, and white blood cell count [14]. GrimAge2 serves as a lifespan biomarker that integrates chronological age, sex, and ten DNA methylation surrogates for cigarette pack-years and plasma protein markers, such as adrenomedullin (ADM), beta-2-microglobulin (B2M), C-reactive protein (CRP), cystatin C, growth differentiation factor-15 (GDF-15), hemoglobin A1c (A1c), leptin, plasminogen activator inhibitor-1 (PAI1), and tissue inhibitor metalloproteinase-1 (TIMP1) [12]. The DNAmTL biomarker estimates telomere length based on DNA methylation patterns [13]. DunedinPoAm measures the pace of biological aging by evaluating morbidity-related biomarkers. This measure was developed using longitudinal data on 18 organ function biomarkers in individuals of the same chronological age, providing a robust indicator of biological aging rate or pace [9].

### Statistical analysis

We employed the R “Survey” package to conduct generalized linear regression models, incorporating NHANES-provided sample weights specific to the epigenetic clock subsample [21]. To explore the relationships of self-rated hearing and vision with each epigenetic age measure, we utilized the *svyglm* function in R, which adjusts for the complex survey design. Our primary model covariates, determined a priori, included chronological age (continuous, in years) and its quadratic term, sex (female vs. male), and self-identified ethnicity/race (non-Hispanic White, Mexican American, other Hispanic, non-Hispanic Black, other race), education level (less than high school, high school diploma/GED, more than high school), occupation (white-collar/professional, white-collar/semi-routine, blue-collar/high-skill, blue-collar/semi-routine, or no work) [22], poverty-to-income ratio (continuous), alcohol intake (abstainer, moderate drinker, heavy drinker), body mass index (BMI [kg/m2]; continuous), smoking status (never, former, current), physical activity (moderate/vigorous activity in the last 30 days: yes vs. no), and self-rated health (good, fair, or poor). For any observed associations involving GrimAge2, the same covariate adjustments were applied in models analyzing relationships with DNAm-predicted blood biomarker components of GrimAge2. To handle missing covariate data, we performed multiple imputation using the *MICE* function in R, generating ten imputed datasets. The results from these datasets were pooled using the *pool* function in R [23]. Acknowledging the strong association of hearing and vision impairments with chronological aging [3, 7], we ran a secondary analysis with the same modeling framework—omitting chronological age adjustments—to compare associations of hearing and vision impairments with chronological age versus those observed with epigenetic age biomarkers.

We performed three sensitivity analyses. To examine the impact of leukocyte proportions on our results, the first sensitivity analysis models were additionally adjusted for estimated leukocyte proportions (B cells, CD4 cells, CD8 cells, NK cells, monocytes, and neutrophils). Second, to assess the possibility of exposure misclassification, we conducted a sensitivity analysis exploring associations of dichotomized self-rated hearing and vision with epigenetic aging. Individuals reporting a little trouble hearing, a lot of trouble hearing, or being deaf were classified as having a hearing impairment, while those reporting good hearing were classified as having no hearing impairment. Similarly, individuals reporting fair, poor, or very poor vision were classified as having a vision impairment, while those reporting good or excellent vision were classified as having no vision impairment. Third, given documented differences between directly measured and DNA methylation–estimated blood proteins [24, 25], we applied the same analytical covariate framework to examine relationships with directly measured CRP available in NHANES. Directly measured CRP was quantified by latex nephelometry in methods previously described [26, 27]. All statistical analyses were conducted using R Version 4.4.1 (R Core Team, Vienna, Austria). Given the small sample sizes in some sensory impairment categories, we used the conservative Bonferroni correction to adjust for multiple comparisons and reduce the risk of type I error. To account for multiple comparisons across the seven epigenetic clocks, statistical significance was set at a Bonferroni-adjusted *P*-value of < 0.007 (0.05/7). *P*-values < 0.05 were considered marginally significant.

## Results

### Study sample characteristics

**Fig. 1:**
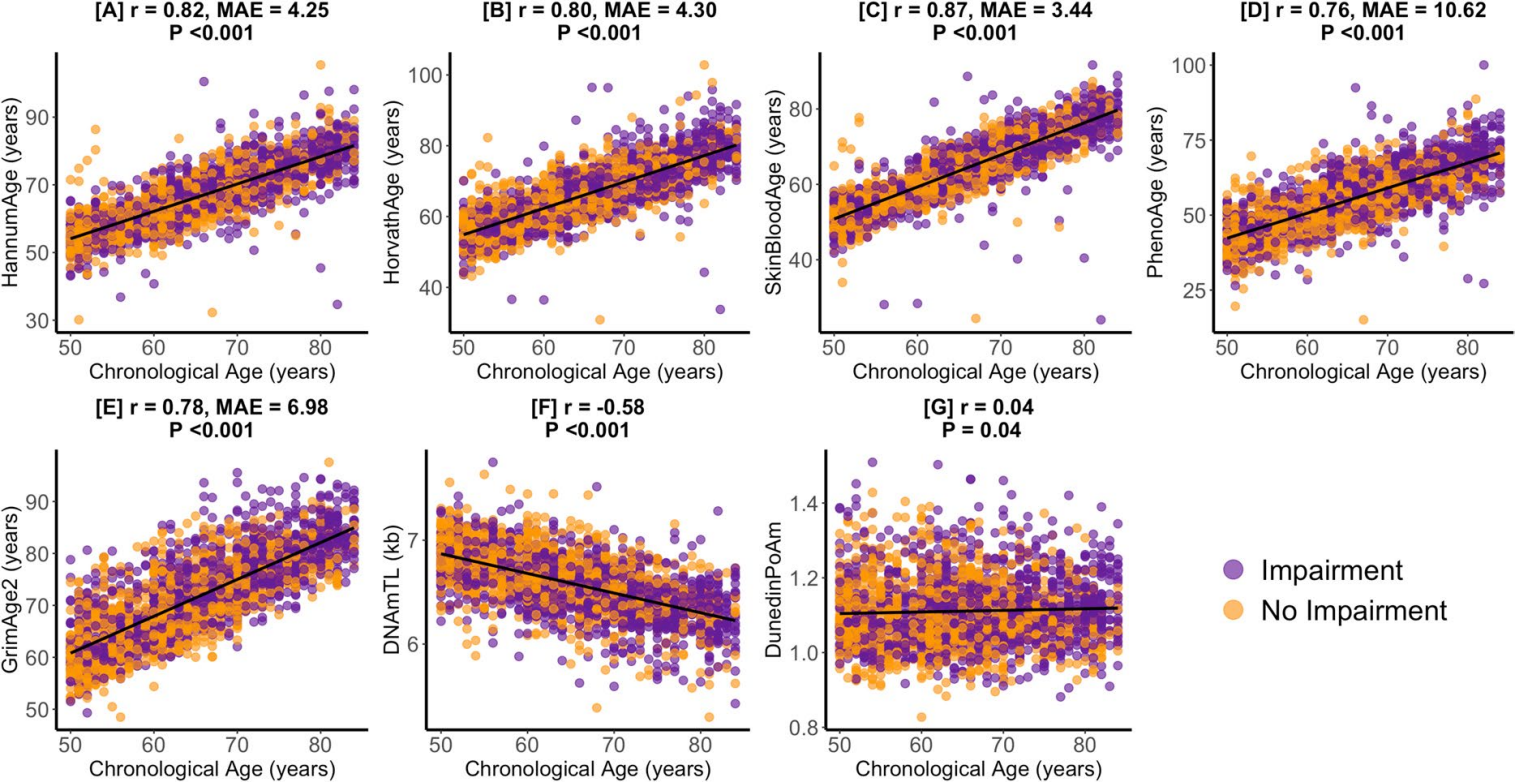
Pearson correlations (*r*) and median absolute error (MAE) of epigenetic age with chronological age. Figure 1 presents the chronological age and epigenetic age correlation coefficients and median absolute errors for the study sample for HannumAge (**A**), HorvathAge (**B**), SkinBloodAge (**C**), PhenoAge (**D**), GrimAge2 (**E**), DNAmTL (**F**), and DunedinPoAm (**G**). Values for participants with an impairment and no impairment in hearing/vision are in purple and orange, respectively

Table 1 presents the unweighted demographic, socioeconomic, and health-related characteristics of the study sample. Participants had a mean (sd) chronological age of 65.1 (9.3) years. Mean (sd) epigenetic age estimates varied across different biomarkers with PhenoAge and SkinBloodAge averages of 54.9 (10.1) years and 63.6 (9.1) years, respectively, both lower than the average chronological age. However, mean HannumAge, HorvathAge, and GrimAge2 were greater than the sample’s average chronological age. Within the study sample, 1% (*n* = 13) reported deafness and 2% (*n* = 47) reported very poor vision. The largest proportion of participants had less than a high school education (45%), worked in blue-collar semi-routine occupations (39%), identified as non-Hispanic White (39%), and were male (51%). With respect to health behaviors, 48% participants were moderate drinkers, 45% were never smokers, and 51% were not physically active. Figure 1 presents the correlations of chronological age with epigenetic biomarkers in the study sample. DNAmTL was negatively correlated with chronological age (*r* = − 0.58, *P* < 0.001). SkinBloodAge exhibited the strongest positive correlation with chronological age (*r* = 0.87, median absolute error [MAE] = 3.44-years, *P* < 0.001).

**Table 1:** Study sample characteristics (*n* = 2344)

Aging variables		
Age (years), mean (sd)		65.1 (9.3)
Epigenetic age/clocks, mean (sd)		
	HannumAge (years)	66.3 (9.2)
	HorvathAge (years)	66.1 (8.6)
	SkinBloodAge (years)	63.6 (9.1)
	PhenoAge (years)	54.9 (10.1)
	GrimAge2 (years)	71.5 (8.4)
	DNAm telomere length (TL) (kb)	6.6 (0.3)
	DunedinPoAm	1.1 (0.1)
Sensory variables		
Hearing, *n* (%)		
	Good	1501 (64)
	Little trouble	652 (28)
	Lot of trouble	178 (7)
	Deaf	13 (1)
Vision*, n* (%)		
	Excellent	496 (21)
	Good	1107 (47)
	Fair	533 (23)
	Poor	134 (6)
	Very poor	47 (2)
	Missing	27 (1)
Demographic variables		
Education, *n* (%)		
	Less than high school	1062 (45)
	High school diploma (including GED)	487 (21)
	More than high school	794 (34)
	missing	1 (0)
Occupation, *n *(%)		
	Blue-collar (high skill)	312 (13)
	Blue-collar (semi-routine)	921 (39)
	White-collar (high skill)	520 (22)
	White-collar (semi-routine)	396 (17)
	Never worked	60 (3)
	Missing	135 (6)
Poverty to income ratio, mean (sd)		
		2.6 (1.6)
	Missing	266
Race/ethnicity category, *n* (%)		
	Mexican American	680 (29)
	Other Hispanic	151 (7)
	Non-Hispanic White	922 (39)
	Non-Hispanic Black	511 (22)
	Other race	80 (3)
Sex, *n* (%)		
	Male	1202 (51)
	Female	1142 (49)
Health behavior variables		
Alcohol intake, *n* (%)		
	Abstainer	1008 (43)
	Moderate drinker	1136 (48)
	Heavy drinker	84 (4)
	Missing	116 (5)
Body mass index (kg/m^2^), mean (sd)		
		28.8 (5.8)
	Missing	83
Self-rated health, *n* (%)		
	Good	1558 (66)
	Fair	598 (26)
	Poor	186 (8)
	Missing	2 (0)
Smoking, *n* (%)		
	Current	374 (16)
	Former	903 (39)
	Never	1063 (45)
	Missing	4 (0)
Physically active, *n* (%)		
	Yes	1147 (49)
	No	1196 (51)
	Missing	1 (0)

### Relationships of self-rated hearing with epigenetic age

After adjusting for covariates, participants who reported being deaf had significantly higher GrimAge2 (*β* = 4.19-years, 95% CI 2.29, 6.09, *P* = 0.004) and DunedinPoAm (*β* = 0.07, 95% CI 0.04, 0.09, *P* = 0.002, Table 2) estimates compared to those reporting good hearing. The GrimAge2 (*β* = 2.09-years, 95% CI 0.04, 4.15, *P* = 0.047) and DunedinPoAm (*β* = 0.03, 95% CI 0.01, 0.05, *P* = 0.02) relationships observed in participants with deafness remained marginally significant after adjusting for estimated leukocyte proportions. No significant associations were observed between other hearing categories and epigenetic aging biomarkers, and dichotomized analyses of self-rated hearing (impaired vs. non-impaired) yielded no significant associations (Table S1).

**Table 2:** Relationships of self-rated hearing with epigenetic aging (*n* = 2344)

	**Main model**		**Leukocyte-adjusted model**	
**Biomarker/hearing**	**Estimate (95% CI)**	***P*-value**	**Estimate (95% CI)**	***P*-value**
**HannumAge**				
Good	ref	-	ref	-
Little trouble	− 0.46 (− 1.59, 0.66)	0.31	− 0.05 (− 0.96, 0.87)	0.89
Lot of trouble	− 0.43 (− 1.75, 0.89)	0.41	− 0.50 (− 1.78, 0.78)	0.33
Deaf	− 2.16 (− 8.64, 4.33)	0.40	− 3.52 (− 9.20, 2.16)	0.16
**HorvathAge**				
Good	ref	-	ref	-
Little trouble	− 0.20 (− 1.16, 0.76)	0.58	0.12 (− 0.85, 1.08)	0.75
Lot of trouble	0.33 (− 1.02, 1.68)	0.53	0.21 (− 1.35, 1.76)	0.73
Deaf	− 0.98 (− 7.06, 5.10)	0.67	− 1.86 (− 7.06, 3.35)	0.37
**SkinBloodAge**				
Good	ref	-	ref	-
Little trouble	− 0.19 (− 1.02, 0.65)	0.56	0.14 (− 0.67, 0.94)	0.65
Lot of trouble	0.13 (− 1.06, 1.31)	0.78	0.08 (− 1.19, 1.35)	0.87
Deaf	− 2.75 (− 8.74, 3.24)	0.27	− 3.47 (− 8.63, 1.7)	0.13
**PhenoAge**				
Good	ref	-	ref	-
Little trouble	− 0.05 (− 1.16, 1.05)	0.90	0.33 (− 0.51, 1.16)	0.33
Lot of trouble	0.46 (− 1.42, 2.33)	0.53	0.05 (− 1.90, 2.00)	0.94
Deaf	3.48 (− 2.86, 9.83)	0.20	1.29 (− 6.09, 8.68)	0.65
**GrimAge2**				
Good	ref	-	ref	-
Little trouble	− 0.12 (− 0.86, 0.61)	0.66	0.10 (− 0.49, 0.70)	0.65
Lot of trouble	0.68 (− 0.44, 1.80)	0.16	0.20 (− 0.71, 1.11)	0.57
Deaf	4.19 (2.29, 6.09)	0.004	2.09 (0.04, 4.15)	0.047
**DNAmTL**				
Good	ref	-	ref	-
Little trouble	0.01 (− 0.04, 0.05)	0.70	− 0.004 (− 0.04, 0.03)	0.78
Lot of trouble	0.02 (− 0.04, 0.08)	0.35	0.02 (− 0.03, 0.07)	0.36
Deaf	− 0.11 (− 0.33, 0.10)	0.21	− 0.09 (− 0.31, 0.13)	0.31
**DunedinPoAm**				
Good	ref	-	ref	-
Little Ttrouble	− 0.01 (− 0.02, 0.01)	0.19	− 0.01 (− 0.02, 0.01)	0.17
Lot of trouble	0.01 (− 0.01, 0.03)	0.16	0.01 (− 0.01, 0.03)	0.34
Deaf	0.07 (0.04, 0.09)	0.002	0.03 (0.01, 0.05)	0.02

Model adjustments: chronological age, chronological age^2^, sex, race/ethnicity, alcohol, BMI, education, occupation, physical activity, PIR, and smoking

*P* < 0.007: statistically significant

*P* < 0.05: marginally significant

Further analysis of GrimAge2 components revealed that participants who reported being deaf had significantly higher estimated levels of TIMP1 (*β* = 459.51, 95% CI 287.00, 632.03 *P* = 0.002) compared to those with good hearing (Table 3). Additionally, deafness was marginally associated with higher estimated levels of ADM (*β* = 10.06, 95% CI 1.76, 18.36, *P* = 0.03), CRP (*β* = 0.34, 95% CI 0.11, 0.56, *P* = 0.01), and cigarette pack-years (*β* = 6.55, 95% CI 2.62, 10.47, *P* = 0.01). Only the relationships with CRP (*β* = 0.23, 95% CI 0.07, 0.40, *P* = 0.02) and pack-years (*β* = 4.35, 95% CI 0.63, 8.06, *P* = 0.03) remained marginally significant after adjusting for estimated leukocyte proportions.

**Table 3:** Relationships of self-rated hearing with GrimAge2 components (*n* = 2344)

	**Main model**		**Leukocyte-adjusted model**	
**Biomarker/hearing**	**Estimate (95% CI)**	***P*-value**	**Estimate (95% CI)**	***P*-value**
**A1c**				
Good	ref	-	ref	-
Little trouble	0.001 (− 0.004, 0.005)	0.72	0.001 (− 0.004, 0.005)	0.75
Lot of trouble	0.001 (− 0.01, 0.01)	0.69	0.002 (− 0.01, 0.01)	0.60
Deaf	0.01 (− 0.01, 0.03)	0.29	0.01 (− 0.01, 0.03)	0.23
**ADM**				
Good	ref	-	ref	-
Little trouble	0.53 (− 2.53, 3.58)	0.65	0.83 (− 1.90, 3.55)	0.44
Lot of trouble	1.92 (− 2.14, 5.98)	0.25	0.57 (− 3.38, 4.53)	0.70
Deaf	10.06 (1.76, 18.36)	0.03	6.85 (− 1.07, 14.77)	0.07
**B2M**				
Good	ref	-	ref	-
Little trouble	4670.28 (− 7490.44, 16,831.01)	0.34	9312.18 (− 4529.85, 23,154.22)	0.13
Lot of trouble	2357.93 (− 19,879.63, 24,595.5)	0.78	− 1129.64 (− 26,959.87, 24,700.59)	0.91
Deaf	32,907.17 (− 62,492, 128,306.3)	0.38	5229.69 (− 100,180.5, 110,639.9)	0.89
**CRP**				
Good	ref	-	ref	-
Little trouble	− 0.01 (− 0.09, 0.06)	0.69	− 0.01 (− 0.07, 0.06)	0.75
Lot of trouble	0.07 (− 0.06, 0.20)	0.20	0.04 (− 0.07, 0.15)	0.35
Deaf	0.34 (0.11, 0.56)	0.01	0.23 (0.07, 0.40)	0.02
**Cystatin C**				
Good	ref	-	ref	-
Little trouble	924.10 (− 3098.35, 4946.55)	0.55	2170.84 (− 907.80, 5249.47)	0.12
Lot of trouble	− 142.57 (− 5205.11, 4919.96)	0.94	− 1955.65 (− 6354.69, 2443.40)	0.28
Deaf	2810.75 (− 13,750.94, 19,372.45)	0.66	− 5995.15 (− 21,399.1, 9408.81)	0.33
**GDF15**				
Good	ref	-	ref	-
Little trouble	− 3.93 (− 24.23, 16.37)	0.61	0.54 (− 17.67, 18.76)	0.94
Lot of trouble	− 3.33 (− 44.76, 38.09)	0.83	− 6.59 (− 45.46, 32.27)	0.66
Deaf	22.77 (− 56.53, 102.06)	0.46	− 0.55 (− 81.40, 80.31)	0.99
**Leptin**				
Good	ref	-	ref	-
Little trouble	− 16.98 (− 264.02, 230.07)	0.85	− 72.31 (− 379.73, 235.12)	0.54
Lot of trouble	− 197.07 (− 659.40, 265.26)	0.29	− 51.87 (− 579.65, 475.90)	0.79
Deaf	− 157.84 (− 1415.99, 1100.31)	0.74	583.16 (− 777.76, 1944.09)	0.29
**Packyears**				
Good	ref	-	ref	-
Little trouble	− 0.53 (− 2.19, 1.13)	0.42	− 0.62 (− 2.35, 1.11)	0.37
Lot of trouble	0.74 (− 1.91, 3.4)	0.47	0.17 (− 2.56, 2.91)	0.86
Deaf	6.55 (2.62, 10.47)	0.01	4.35 (0.63, 8.06)	0.03
**PAI1**				
Good	ref	-	ref	-
Little trouble	54.85 (− 429.32, 539.03)	0.76	95.11 (− 360.31, 550.52)	0.59
Lot of trouble	77.52 (− 762.49, 917.52)	0.81	− 22.83 (− 867.94, 822.28)	0.94
Deaf	1735.75 (− 1075.08, 4546.59)	0.16	1516.06 (− 1319.48, 4351.59)	0.21
**TIMP1**				
Good	ref	-	ref	-
Little trouble	− 25.12 (− 133.86, 83.62)	0.55	32.07 (− 71.28, 135.43)	0.43
Lot of trouble	111.93 (− 29.07, 252.92)	0.09	48.32 (− 126.54, 223.18)	0.48
Deaf	459.51 (287.00, 632.03)	0.002	173.62 (− 313.02, 660.26)	0.37

Model adjustments: chronological age, chronological age^2^, sex, race/ethnicity, alcohol, BMI, education, occupation, physical activity, PIR, and smoking

*P* < 0.007: statistically significant

*P* < 0.05: marginally significant

###  Relationships of self-rated vision with epigenetic age

We did not observe any statistically or marginally significant relationships of self-rated vision with any epigenetic age biomarker (Table 4). Similarly, analyses using less discrete categorizations of self-rated vision did not yield significant associations (Table S2).

**Table 4:** Relationships of self-rated vision with epigenetic aging (*n* = 2317)

	**Main model**		**Leukocyte-adjusted model**	
**Biomarker/hearing**	**Estimate (95% CI)**	***P*-value**	**Estimate (95% CI)**	***P*-value**
**HannumAge**				
Excellent	ref	-	ref	-
Good	0.09 (− 1.35, 1.53)	0.85	− 0.03 (− 1.53, 1.48)	0.96
Fair	− 0.44 (− 2.01, 1.14)	0.43	− 0.73 (− 2.61, 1.14)	0.29
Poor	0.35 (− 1.88, 2.57)	0.65	0.61 (− 1.56, 2.78)	0.43
Very poor	− 0.61 (− 3.03, 1.80)	0.47	− 0.27 (− 3.89, 3.36)	0.83
**HorvathAge**				
Excellent	ref	-	ref	-
Good	0.62 (− 0.91, 2.15)	0.28	0.55 (− 0.99, 2.09)	0.33
Fair	0.08 (− 1.50, 1.66)	0.88	− 0.22 (− 1.96, 1.51)	0.70
Poor	0.64 (− 1.62, 2.90)	0.42	0.86 (− 1.02, 2.75)	0.23
Very poor	1.08 (− 3.33, 5.48)	0.48	1.18 (− 4.30, 6.65)	0.53
**SkinBloodAge**				
Excellent	ref	-	ref	-
Good	0.16 (− 1.23, 1.54)	0.74	0.11 (− 1.34, 1.57)	0.82
Fair	0.17 (− 1.31, 1.66)	0.73	− 0.08 (− 1.65, 1.49)	0.87
Poor	1.48 (− 0.49, 3.45)	0.10	1.77 (− 0.31, 3.85)	0.07
Very poor	− 0.40 (− 3.07, 2.26)	0.65	− 0.15 (− 4.12, 3.83)	0.91
**PhenoAge**				
Excellent	ref	-	ref	-
Good	0.57 (− 0.98, 2.13)	0.32	0.45 (− 1.17, 2.08)	0.43
Fair	0.21 (− 1.44, 1.86)	0.70	0.03 (− 1.82, 1.88)	0.96
Poor	0.81 (− 1.79, 3.40)	0.39	0.91 (− 1.95, 3.77)	0.38
Very poor	1.50 (− 1.92, 4.92)	0.25	1.67 (− 2.61, 5.95)	0.30
**GrimAge2**				
Excellent	ref	-	ref	-
Good	0.38 (− 0.37, 1.14)	0.20	0.33 (− 0.46, 1.13)	0.27
Fair	0.61 (− 0.62, 1.84)	0.21	0.53 (− 0.58, 1.64)	0.22
Poor	1.14 (− 0.74, 3.03)	0.15	1.04 (− 0.99, 3.08)	0.20
Very poor	1.21 (− 0.87, 3.30)	0.16	1.28 (− 1.41, 3.98)	0.22
**DNAmTL**				
Excellent	ref	-	ref	-
Good	0.01 (− 0.04, 0.07)	0.48	0.02 (− 0.04, 0.07)	0.43
Fair	− 0.01 (− 0.09, 0.07)	0.64	0.001 (− 0.07, 0.07)	0.95
Poor	− 0.04 (− 0.15, 0.08)	0.36	− 0.03 (− 0.13, 0.07)	0.38
Very poor	0.01 (− 0.13, 0.15)	0.78	0.002 (− 0.16, 0.16)	0.97
**DunedinPoAm**				
Excellent	ref	-	ref	-
Good	0.002 (− 0.01, 0.02)	0.67	0.002 (− 0.01, 0.01)	0.59
Fair	− 0.01 (− 0.04, 0.02)	0.46	− 0.004 (− 0.03, 0.02)	0.55
Poor	0.02 (− 0.02, 0.05)	0.23	0.01 (− 0.02, 0.05)	0.42
Very poor	− 0.03 (− 0.09, 0.03)	0.19	− 0.02 (− 0.07, 0.03)	0.25

Model adjustments: chronological age, chronological age^2^, sex, race/ethnicity, alcohol, BMI, education, occupation, physical activity, PIR, and smoking

*P* < 0.007: statistically significant

*P* < 0.05: marginally significant

### Relationships of self-rated hearing and vision with chronological age

To contextualize our findings on epigenetic age, we examined the associations between self-rated hearing and vision and chronological age. In fully adjusted models, participants who reported a little trouble (*β* = 2.67-years, 95% CI 1.17, 4.16, *P* = 0.005) or a lot of trouble hearing (*β* = 6.30-years, 95% CI 3.56, 9.04, *P* = 0.002) were chronologically older than participants who reported good hearing (Table 5). These associations were slightly attenuated but remained statistically significant after adjusting for estimated leukocyte proportions. In contrast, no significant association was observed between deafness and chronological age. Consistent with our findings for epigenetic aging biomarkers, self-rated vision did not exhibit any statistically or marginally significant associations with chronological age (Table 5).

**Table 5:** Relationships of self-rated hearing and vision with chronological age

	**Main model**		**Leukocyte-adjusted model**	
	**Estimate (95% CI)**	***P*-value**	**Estimate (95% CI)**	***P*-value**
**Hearing (*n* = 2344)**				
Good	ref	-	ref	-
Little trouble	2.67 (1.17, 4.16)	0.005	2.65 (1.25, 4.05)	0.004
Lot of trouble	6.30 (3.56, 9.04)	0.002	5.42 (2.89, 7.95)	0.002
Deaf	− 0.37 (− 6.37, 5.64)	0.88	− 0.81 (− 5.49, 3.86)	0.68
**Vision (*n* = 2317)**				
Excellent	ref	-	ref	-
Good	0.60 (− 1.42, 2.63)	0.47	0.46 (− 1.65, 2.57)	0.60
Fair	1.19 (− 1.39, 3.77)	0.28	0.82 (− 1.64, 3.27)	0.42
Poor	1.27 (− 3.24, 5.78)	0.49	1.45 (− 2.94, 5.83)	0.43
Very poor	3.95 (− 1.85, 9.75)	0.14	3.52 (− 1.12, 8.15)	0.11

Model adjustments: sex, race/ethnicity, alcohol, BMI, education, occupation, physical activity, PIR, and smoking

*P* < 0.007: statistically significant

*P* < 0.05: marginally significant

### Relationships of self-rated hearing with directly measured CRP

Although GrimAge2 component estimated CRP and directly measured CRP were moderately correlated (*r* = 0.29, *P* < 0.001), self-rated hearing was not significantly or marginally associated with directly measured CRP in fully adjusted models (Table S3).

## Discussion

In this analysis of a nationally representative cross-sectional sample of U.S. adults aged 50–84 years, we examined the associations of self-rated hearing and vision with epigenetic aging biomarkers. By focusing on self-rated sensory function, our study extends and complements previous research demonstrating that epigenetic aging biomarkers are sensitive to objective measures of sensory impairment [8, 16, 17]. To our knowledge, this is the first study to explore these relationships using self-rated sensory assessments, providing novel insights into the potential role of perceived sensory impairments in biological aging. Our findings demonstrated that participants who reported being deaf exhibited higher levels of GrimAge2 and DunedinPoAm estimates compared to those who reported good hearing, suggesting an association between profound hearing loss and accelerated epigenetic aging. Additionally, deaf participants exhibited significantly higher estimated levels of DNAm TIMP1 and marginally higher levels of DNAm ADM, DNAm CRP, and pack-years, further implicating inflammatory and metabolic pathways in hearing loss-related aging processes. In contrast, no statistically significant associations were observed between self-rated vision and epigenetic aging biomarkers, suggesting potential differences in the biological underpinnings of auditory and visual aging or limitations in the sensitivity of self-reported vision measures for detecting age-related epigenetic changes.

We hypothesized that greater sensory impairment would be associated with greater epigenetic aging, and this was observed for self-rated deafness. Moreover, our findings align with results from the Baltimore Longitudinal Study of Aging, which reported that higher speech frequency pure tone average, an indicator of worse auditory function, was associated with greater GrimAge and DunedinPoAm estimates but not with older epigenetic aging biomarkers [16]. This lack of association with earlier generation biomarkers is likely due to DunedinPoAm and GrimAge2 incorporating clinical variables that enhance their sensitivity to human disease processes [9, 12].

We expand on prior literature by identifying TIMP1 as a GrimAge2 component associated with deafness. TIMP1 is a multifunctional protein that inhibits matrix metalloproteinases (MMPs), which regulate extracellular matrix degradation [28]. Beyond its structural role, TIMP1 has been implicated in inflammatory regulation, particularly in autoimmune diseases. Elevated circulating levels of TIMP1, as observed in participants reporting deafness, have been linked to neuroinflammation [29], which can contribute to auditory dysfunction and hearing loss [30, 31]. Consistent with this, a study conducted in New York, U.S., found that patients with autoimmune inner ear disease had significantly higher blood levels of TIMP1 than healthy controls [32]. Additionally, studies in rat models have implicated TIMP1 upregulation in noise-induced ear damage, further supporting its role in hearing-related pathophysiology [33].

The relationship of TIMP1 with inflammation may also explain why we observe marginal associations of higher DNAm estimates of smoking pack-years, CRP, and ADM levels in deaf participants. ADM is a vasoactive peptide that can be elevated in inflammation [34]. While vasoactive substances have been considered as therapies for sudden sensorineural hearing loss [35], the exact relationship between ADM and hearing loss requires further study. Additionally, smoking has been previously associated with hearing loss, partially through its contributions to systemic inflammation, while CRP serves as a general marker of inflammation [36–38]. Given these connections, it is possible that inflammation serves as a mediating factor in the observed associations, which may also explain why our results were attenuated after adjusting for estimated leukocyte proportions.

It is noteworthy that our DNAm-based CRP measure demonstrated a stronger association with auditory function than directly measured serum CRP. This finding is consistent with prior research in the Lothian Birth Cohort, which reported that DNAm-based CRP estimates exhibited significantly stronger associations with brain health compared to serum CRP levels [39]. This may further suggest that epigenetic biomarkers may capture chronic inflammatory processes more effectively than traditional biomarkers, reinforcing their potential role in aging and sensory decline research.

Notably, we did not observe any marginal or significant associations of self-rated vision with epigenetic aging. Several factors may explain this lack of an association, including the possibility that epigenetic aging biomarkers are more sensitive to objective measures of vision, as previously reported, and may not capture subjective assessments as effectively [8, 17]. Another key difference may stem from how the questions were structured in the NHANES questionnaire. Specifically, participants were asked to evaluate their auditory function independent of hearing aids but were instructed to assess their vision considering the use of corrective aids such as glasses or contact lenses. It is possible that if participants had been asked to rate their vision without visual aids, we would have observed a different distribution of self-rated visual function, which might have yielded stronger associations with epigenetic age measures. Future studies should consider revising the framing of self-reported vision assessments to better capture their relationships with biological aging.

Additionally, while we adjusted for general health status, our models did not account for specific age-related ocular diseases, such as age-related macular degeneration, cataracts, diabetic retinopathy, and open-angle glaucoma, all of which contribute to visual impairment in aging populations [40]. Future research examining epigenetic aging in these specific disease contexts could provide greater insight into the role of biological aging in vision loss.

Although our findings for self-rated vision were largely null, the importance of considering disease-specific contexts is reinforced by the differences we observed between self-rated hearing with chronological versus epigenetic aging. In models using chronological age, having a little or a lot of trouble hearing was associated with older age, whereas deafness was not. In this study, we did not have data to explain the etiology of the participants’ deafness, but this pattern underscores the potential for epigenetic aging to serve as a more sensitive marker of morbidity compared to chronological age, as suggested in prior research [18]. Further exploration in disease-specific populations could clarify whether certain sensory impairments are more closely linked to biological aging processes than others.

Our study has several notable strengths, particularly the use of DNAm-based biomarkers to examine associations between self-rated auditory and visual function and epigenetic aging. Nonetheless, certain limitations should be considered. First, we focused on self-rated measures of sensory function, and while these provide valuable insights into perceived impairments, they may not fully capture the biological processes underlying sensory decline. Relationships with objective measures were beyond the scope of this analysis but warrant further investigation, as they may reveal patterns of association with epigenetic aging. Second, the sample sizes for greater impairments like deafness were relatively small. Although we performed numerous sensitivity analyses with consistent results, we recommend future studies with larger sample sizes of impaired individuals to better characterize these relationships. Third, while NHANES provides extensive demographic and lifestyle data, some participants had missing information. To address this, we implemented multiple imputation, reducing potential bias associated with missing covariates. Fourth, the cross-sectional design of our study precludes an assessment of longitudinal trends, which are essential for understanding when impairments occurred, the progression of sensory impairments, and their relationship with epigenetic aging. It is possible that epigenetic aging increases the risk of deafness, that deafness contributes to accelerated epigenetic aging, or that both processes influence each other. Future research incorporating longitudinal data is needed to capture these dynamic relationships over time. Fifth, the data analyzed in this study were collected approximately two decades ago, raising potential concerns about generalizability to the current U.S. population, given advancements in healthcare access, interventions, and environmental exposures. However, these remain the most recent DNAm data available within NHANES, making them an essential resource for studying epigenetic aging at the population level. Lastly, although we adjusted for key covariates in our models, the possibility of residual or unmeasured confounding, from factors like noise exposure or underlying disease processes, cannot be entirely ruled out. Despite these limitations, our study provides novel insights into the relationships of sensory function with biological aging. By leveraging a nationally representative sample, these findings can inform future research incorporating newer epigenetic datasets and highlight the importance of examining sensory function in the context of aging-related processes.

## Conclusion

In conclusion, this study of U.S. adults aged 50–84 years reports that self-rated deafness was associated with accelerated epigenetic aging, as measured by GrimAge2 and DunedinPoAm, along with elevated estimated levels of the GrimAge2-predicted protein TIMP1. These findings suggest that epigenetic aging biomarkers may capture underlying biological processes linked to hearing loss, including inflammatory and extracellular matrix remodeling pathways. Again, the sample sizes for more severe impairments, such as deafness, were relatively small, which raises the potential for outlier driven findings and limited generalizability. However, our extensive sensitivity analyses yielded consistent results, suggesting robustness. Nonetheless, we recommend future studies with larger samples of individuals with significant impairments to more precisely characterize these associations. If validated, epigenetic aging measures could serve as valuable tools for predicting sensory impairments and elucidating their role in broader aging processes. Future research integrating longitudinal epigenetic changes and mechanistic studies will be critical for determining whether epigenetic biomarkers can enhance early detection and intervention strategies for sensory decline in aging populations.

## Supplementary Information

Below is the link to the electronic supplementary material.

Supplementary file1 (DOCX 36 KB)

## Data Availability

The datasets analyzed in the current study are available from the NHANES website.

## Declarations

### Institutional clearance

Not applicable. All authors reviewed and approved the final manuscript.
