# Supplementary material for: Sensory impairments and epigenetic aging: insights from self-rated hearing and vision in United States adults
Source: GeroScience. 2025 May 23;48(1):1037–50. doi: 10.1007/s11357-025-01706-6 (PMC12972156; doi:10.1007/s11357-025-01706-6)
Supplement: Supplementary file 1 — Supplementary file1 (DOCX 36 KB) [file 11357_2025_1706_MOESM1_ESM.docx]

| **Table S1. Relationships of Self-Rated Hearing in Less Discrete Categories with Epigenetic Aging (n = 2344)** | | | | |
| --- | --- | --- | --- | --- |
|  | **Main Model** | | **Leukocyte-Adjusted Model** | |
| **Biomarker/Hearing** | **Estimate (95% CI)** | ***P*-value** | **Estimate (95% CI)** | ***P*-value** |
| **HannumAge** |  |  |  |  |
| No Impairment | ref | - | ref | - |
| Impairment | -0.49 (-1.38, 0.40) | 0.23 | -0.18 (-0.94, 0.58) | 0.58 |
|  |  |  |  |  |
| **HorvathAge** |  |  |  |  |
| No Impairment | ref | - | ref | - |
| Impairment | -0.13 (-1.00, 0.75) | 0.73 | 0.10 (-0.74, 0.94) | 0.78 |
|  |  |  |  |  |
| **SkinBloodAge** |  |  |  |  |
| No Impairment | ref | - | ref | - |
| Impairment | -0.18 (-0.95, 0.59) | 0.58 | 0.07 (-0.66, 0.79) | 0.83 |
|  |  |  |  |  |
| **PhenoAge** |  |  |  |  |
| No Impairment | ref | - | ref | - |
| Impairment | 0.09 (-0.83, 1.01) | 0.82 | 0.30 (-0.39, 0.98) | 0.33 |
|  |  |  |  |  |
| **GrimAge2** |  |  |  |  |
| No Impairment | ref | - | ref | - |
| Impairment | 0.08 (-0.53, 0.69) | 0.76 | 0.15 (-0.32, 0.62) | 0.46 |
|  |  |  |  |  |
| **DNAmTL** |  |  |  |  |
| No Impairment | ref | - | ref | - |
| Impairment | 0.01 (-0.03, 0.04) | 0.65 | -0.001 (-0.03, 0.03) | 0.91 |
|  |  |  |  |  |
| **DunedinPoAm** |  |  |  |  |
| No Impairment | ref | - | ref | - |
| Impairment | -0.003 (-0.01, 0.01) | 0.48 | -0.004 (-0.01, 0.01) | 0.35 |
|  |  |  |  |  |
| **Model Adjustments:** chronological age, chronological age^2^, sex, race/ethnicity, alcohol, BMI, education, occupation, physical activity, PIR, and smoking. No Impairment: Good hearing. Impairment: Little/Lot Trouble hearing & Deaf.  *P* <0.007: statistically significant  *P* <0.05: marginally significant | | | | |

| **Table S2. Relationships of Self-Rated Vision in Less Discrete Categories with Epigenetic Aging (n = 2317)** | | | | |
| --- | --- | --- | --- | --- |
|  | **Main Model** | | **Leukocyte-Adjusted Model** | |
| **Biomarker/Hearing** | **Estimate (95% CI)** | ***P*-value** | **Estimate (95% CI)** | ***P*-value** |
| **HannumAge** |  |  |  |  |
| No Impairment | ref | - | ref | - |
| Impairment | -0.38 (-1.08, 0.32) | 0.23 | -0.47 (-1.3, 0.37) | 0.22 |
|  |  |  |  |  |
| **HorvathAge** |  |  |  |  |
| No Impairment | ref | - | ref | - |
| Impairment | -0.20 (-0.95, 0.55) | 0.53 | -0.35 (-1.11, 0.42) | 0.31 |
|  |  |  |  |  |
| **SkinBloodAge** |  |  |  |  |
| No Impairment | ref | - | ref | - |
| Impairment | 0.24 (-0.36, 0.85) | 0.35 | 0.14 (-0.59, 0.88) | 0.31 |
|  |  |  |  |  |
| **PhenoAge** |  |  |  |  |
| No Impairment | ref | - | ref | - |
| Impairment | -0.02 (-0.89, 0.86) | 0.96 | -0.05 (-1, 0.89) | 0.31 |
|  |  |  |  |  |
| **GrimAge2** |  |  |  |  |
| No Impairment | ref | - | ref | - |
| Impairment | 0.47 (-0.24, 1.18) | 0.15 | 0.43 (-0.18, 1.03) | 0.13 |
|  |  |  |  |  |
| **DNAmTL** |  |  |  |  |
| No Impairment | ref | - | ref | - |
| Impairment | -0.03 (-0.06, 0.01) | 0.12 | -0.02 (-0.05, 0.02) | 0.27 |
|  |  |  |  |  |
| **DunedinPoAm** |  |  |  |  |
| No Impairment | ref | - | ref | - |
| Impairment | -0.01 (-0.02, 0.01) | 0.39 | -0.005 (-0.02, 0.01) | 0.42 |
|  |  |  |  |  |
| **Model Adjustments:** chronological age, chronological age^2^, sex, race/ethnicity, alcohol, BMI, education, occupation, physical activity, PIR, and smoking. No Impairment: Good, Excellent vision. Impairment: Fair, Poor, Very Poor vision.  *P* <0.007: statistically significant  *P* <0.05: marginally significant | | | | |

| **Table S3. Relationships of Self-Rated Hearing with Directly Measured CRP (n = 2344)** | | | | |
| --- | --- | --- | --- | --- |
|  | **Main Model** | | **Leukocyte-Adjusted Model** | |
|  | **Estimate (95% CI)** | ***P*-value** | **Estimate (95% CI)** | ***P*-value** |
| **Hearing (n = 2344)** |  |  |  |  |
| Good | ref | - | ref | - |
| Little Trouble | -0.14 (-0.32, 0.04) | 0.10 | -0.14 (-0.31, 0.02) | 0.08 |
| Lot of Trouble | -0.10 (-0.36, 0.16) | 0.35 | -0.12 (-0.36, 0.11) | 0.22 |
| Deaf | 0.26 (-0.42, 0.95) | 0.34 | 0.10 (-0.56, 0.76) | 0.69 |
|  |  |  |  |  |
| **Model Adjustments:** chronological age, chronological age^2^, sex, race/ethnicity, alcohol, BMI, education, occupation, physical activity, PIR, and smoking.  *P* <0.007: statistically significant  *P* <0.05: marginally significant | | | | |

n = 27

missing vision data

n = 2,532

participants in the NHANES 1999-2002 epigenetics biomarkers dataset.

n = 2,402

n = 130

coded as 85 years with unknown exact chronological age.

n = 56

with mismatch in DNA methylation predicted and self-reported sex.

n = 2,346

n = 2

missing hearing data

n = 2,344

final hearing study sample

n = 2,317

final vision study sample

**Figure S1. Participant Analysis Flow Chart. Figure S1** presents a flowchart describing how the final study sample was achieved.
